# Large Language Models in Colorectal Cancer Care and Clinical Decision Support: Systematic Review

**DOI:** 10.2196/89862

**Published:** 2026-05-21

**Authors:** Jinglei Tian, Qifeng Lou, Xue Wang, Hangying Xu, Huiting Mei, Yanli Yu

**Affiliations:** 1 Zhejiang Chinese Medical University, Zhejiang Chinese Medical University, Hangzhou, China, Hangzhou, Zhejiang, China; 2 Department of Gastroenterology, Hangzhou First People's Hospital, Hangzhou, China, Hangzhou, Zhejiang, China, 86 15267498545

**Keywords:** artificial intelligence, large language models, colorectal cancer, gastroenterology, systematic review, PRISMA

## Abstract

**Background:**

Colorectal cancer (CRC) is a leading cause of cancer morbidity and mortality worldwide. The complexity of guideline-concordant care and unstructured clinical data has driven demand for decision-support tools. Large language models (LLMs) show promise for processing clinical data and patient–provider communication, yet evidence is fragmented, and a CRC-specific synthesis across the full care continuum is lacking.

**Objective:**

This systematic review evaluates the current applications, performance determinants, and clinical implications of LLMs across the continuum of CRC care.

**Methods:**

Following PRISMA (Preferred Reporting Items for Systematic Reviews and Meta-Analyses), we searched 6 databases (PubMed, Embase, Web of Science, Scopus, CINAHL, Cochrane) through April 1, 2026. Eligible studies were peer-reviewed original investigations of LLMs on CRC tasks with extractable outcomes; reviews, editorials, and abstracts were excluded. Two reviewers assessed quality with QUADAS-2 (Quality Assessment of Diagnostic Accuracy Studies-2), PROBAST (prediction model risk of bias assessment tool), and ROBINS-I (Risk of Bias in Nonrandomized Studies - of Interventions). Data on model types, applications, prompts, input/output formats, and outcomes were analyzed descriptively, with narrative synthesis per synthesis without meta-analysis (SWiM) guidelines.

**Results:**

Of 8880 records, 37 studies met inclusion criteria (2023‐2026), mostly from China and the United States, with GPT series most frequently evaluated. Overall risk of bias was low in 10/37 studies (27.0%), moderate in 14/37 (37.8%), unclear in 7/37 (18.9%), and high or serious in 6/37 (16.2%). Problematic domains included outcome measurement, intervention classification, patient selection, and lack of blinded assessment. LLMs showed utility in automating data extraction from clinical texts, supporting patient education, aiding diagnosis, and assisting clinical decision-making, with emerging visual interpretation and multimodal capacities. Domain-specific and multimodal models showed advantages over general-purpose models in certain tasks. Performance was significantly influenced by prompt design, from zero-shot queries to fine-tuning. Despite efficiency and outcome benefits, challenges persist regarding methodological quality, data privacy, and generalizability.

**Conclusions:**

This review provides an integrative framework synthesizing evidence across study designs and LLM categories in CRC care. Unlike prior reviews addressing gastroenterology broadly or limited to one design, it covers the full CRC continuum and, for the first time, comparatively evaluates general-purpose, domain-specific, and multimodal LLMs, clarifying how prompt engineering and heterogeneous metrics shape outcomes. Although findings support LLMs’ clinical potential, results must be interpreted cautiously, given low overall evidence quality. Most studies lacked safeguards against bias—blinded assessment, confounder adjustment, or prospective multicenter validation. Substantial heterogeneity across tasks, LLM types, prompts, reference standards, and outcomes means reported advantages cannot be generalized. Future work should prioritize real-world integration via prospective multicenter validation, robust privacy frameworks, and rigorous human oversight. Amid rising global CRC burden and health care disparities, this review informs clinical translation, equitable scaling, and policy on LLM deployment.

## Introduction

Colorectal cancer (CRC) is the third most commonly diagnosed malignancy and the second leading cause of cancer-related mortality worldwide, with incidence projected to rise substantially through 2050 [1]. Contemporary CRC care spans a long continuum: risk stratification, screening, endoscopic and histopathological diagnosis, multidisciplinary treatment, and long-term surveillance, in which each stage generates dense, largely unstructured clinical text and requires time-sensitive, guideline-concordant decisions [2]. This labor-intensive process is time-consuming and error-prone due to visual fatigue and information gaps inherent in voluminous clinical notes, pulling clinicians from direct patient care and straining both providers and institutional resources [3,4]. Within this context, large language models (LLMs) built on the Transformer architecture have emerged as a candidate interface between complex clinical text and decision support [5]. Compared with conventional clinical decision-support and patient education modalities, LLMs offer several distinct advantages: automated extraction and processing of large-scale clinical follow-up records [6], real-time responses to patient inquiries regarding CRC symptoms and prevention [7], guidance for geographically tailored screening strategies [8], and enhanced adherence to clinical quality improvement initiatives [5], less constrained by outpatient scheduling or geographic disparities in health care resource distribution [9]. This approach conserves clinician time and reduces operational costs while simultaneously improving the accessibility, flexibility, and scalability of CRC-related health information for patients [10].

Against this backdrop, research on LLMs in CRC has expanded rapidly between 2024 and 2026, spanning the entire care continuum. In screening and early detection, GPT-4 and its successors have been evaluated for risk-stratified counseling and family-history triage for hereditary CRC syndromes [11,12], while multiple studies have also explored the clinical utility of LLM-based tools, notably ChatGPT (OpenAI), for preoperative screening consultations and postoperative surveillance monitoring in CRC patients [13,14]. In endoscopy, LLMs have been applied to automate colonoscopy report generation [15,16]. In pathology, LLMs have been used to extract tumor–node–metastasis (TNM) descriptors and microsatellite instability status [17,18]. Therapeutic decision support has emerged as a particularly active area, with LLM recommendations benchmarked against multidisciplinary tumor board consensus [19,20]. The accelerating volume of these publications makes a focused, structured synthesis both timely and necessary.

Nevertheless, digital health models are not without limitations, including technically inaccurate outputs attributable to hallucinations [21], quality assurance concerns in complex diagnostic and therapeutic recommendations [22], and challenges related to model bias, limited generalizability, and the absence of physician empathy [23]. The emerging literature also reflects substantial heterogeneity, with findings that vary across studies. Model selection is one key factor [24]. Published studies have compared various general-purpose and medically fine-tuned models, with consistent reports distinguishing the performance of GPT-4-class and domain-tuned models from that of earlier or smaller backbones in oncology evaluations, while open-source models offer data-privacy advantages but display variable accuracy across CRC tasks [24,25]. Equally consequential is the choice of prompt engineering strategy: zero-shot prompting, few-shot prompting, chain-of-thought reasoning, retrieval-augmented generation (RAG), and guideline-grounded prompting yield markedly different accuracy on identical CRC questions [14,25-28], with several studies reporting accuracy gains when few-shot or RAG approaches replace naive zero-shot baselines [27,29,30]. Additional sources of heterogeneity include differences in evaluation rubrics, question framing, and prompt language [31,32]. Consequently, 2 studies addressing apparently similar questions can reach opposing conclusions. Amini et al [13] assessed the clinical utility of freely available LLMs for colonoscopy surveillance interval recommendations across diverse settings, finding insufficient accuracy and notable limitations. In contrast, Chang et al [14], using the more capable GPT-4 model and a guideline-anchored expert panel as reference, concluded that ChatGPT-4 exhibited accuracy comparable to professional gastroenterologists.

Within the gastroenterological domain, several reviews have mapped LLM applications. Gong conducted a systematic review of LLMs in gastroenterology and gastrointestinal endoscopy, categorizing applications into knowledge-based response evaluation and document automation, with most studies focusing on GPT-series models [9]. Omar et al [15] reviewed 57 natural language processing (NLP) and LLM studies in gastroenterology and hepatology, confirming improved data extraction from electronic health records (EHRs) but noting persistent challenges in integrating these tools into routine clinical practice. Furthermore, a recent systematic review in lung cancer identified critical methodological limitations in primary LLM studies, notably a reliance on retrospective data and unclear risk of bias [33]. Given the fundamental differences in oncology protocols, the specific, multi-stage clinical trajectory of CRC, spanning distinct endoscopic, pathological, and surgical phases, necessitates an isolated, disease-specific appraisal to objectively evaluate LLM viability. However, a conspicuous gap remains: no systematic review has comprehensively evaluated the evidence for LLM applications specifically within the CRC domain. In particular, the information quality of LLM outputs across the full CRC care continuum has been insufficiently addressed in prior systematic reviews. Compounding this limitation, although recent studies have demonstrated that LLMs can achieve clinician-level performance in specific clinical tasks, substantial heterogeneity in model selection, prompt engineering strategies, and evaluation metrics precludes generalizable conclusions [34,35].

Accordingly, this systematic review aims to evaluate the performance of different LLM categories across the full CRC care continuum, address evidence gaps arising from fragmented research practices, and provide a foundation for future research and clinical translation, covering use cases, model types, optimization strategies, limitations, and future directions. Specifically, this review seeks to (1) map LLM applications across the principal clinical domains of CRC management; (2) compare general-purpose, domain-specific, and multimodal LLMs under different prompt engineering and fine-tuning strategies; (3) classify included studies according to their research design and apply corresponding quality appraisal tools to appraise the credibility of individual studies.

## Methods

### Eligibility Criteria

The eligibility criteria for this review were established according to the PICOS (Population, Intervention, Comparison, Outcome, Study design) framework, as detailed in Table 1.

**Table 1.** PICOS (Population, Intervention, Comparison, Outcome, Study design) eligibility criteria.

Criteria	Definition
Participants	General population or patients with CRC.
Intervention	Artificial Intelligence, specifically LLM^a^ applied in CRC^b^ management. These may be applications used by patients or health care providers for auxiliary diagnosis, information extraction, knowledge-based question answering, treatment decision-making, predictive modeling, or scientific research. LLMs are advanced AI^c^ systems designed to process complex clinical data, support decision-making, and enable effective communication.
Control	Control (applicable exclusively to comparative study designs): Standard clinical evaluation by health care professionals or conventional non-LLM computational algorithms. Studies without a control group were eligible for inclusion if the other criteria were met.
Outcomes	Outcome measures included: Clinical and performance effectiveness (eg, Accuracy, *F*_1_-score, area under the curve, sensitivity, concordance rate) and qualitative/utility measures (eg, response completeness, clarity, comprehensiveness, guideline adherence).
Study types	All study types were considered (eg, exploratory or comparative designs) so long as the original research concept was implemented and tested regarding LLMs and CRC. Nonoriginal research such as books, book chapters, letters, reviews, and conference proceedings were excluded.
Other	Studies were restricted to English language only articles.

a LLM: large language model.

b CRC: colorectal cancer.

c AI: artificial intelligence.

Discrepancies were resolved by discussion, with arbitration by a third reviewer. This review was conducted following PRISMA (Preferred Reporting Items for Systematic Reviews and Meta-Analyses) 2020 [36], with search reporting per PRISMA-S [37] and narrative synthesis per SWiM guidelines [38].

### Information Sources

Relevant studies were identified by systematically searching 6 electronic databases: PubMed, Web of Science, Embase, Cochrane Library, CINAHL, and Scopus (search cutoff date: April 1, 2026). Each database was searched individually; no multi-database searching on a single platform was performed. No published search filters (eg, validated study design filters) were applied to any database search.

### Search Strategy

The search strategy combined Medical Subject Headings (MeSH and EMTREE) and free-text keywords related to CRC and LLMs. These terms were adapted for each database to maximize retrieval sensitivity. Key terms included: “colonic neoplasms,” “colorectal cancer*,” “large language models,” “artificial intelligence,” “LLM,” “GPT,” “ChatGPT,” “Claude,” “Gemini,” and “LLaMA.” The search process followed the PRISMA Search Strategy Extension [20]. The complete search strategy, including specific search queries, applied limits, and the number of records retrieved from each database, is provided in Multimedia Appendix 1. The initial search was established and updated through April 1, 2026, to capture the most recent publications prior to data synthesis.

Regarding the PRISMA-S checklist, certain items were not applicable to our methodology: study registries and regulatory databases were not searched, as research on LLMs in CRC is generally not registered as clinical trials; gray literature, institutional websites, conference proceedings, and preprint servers were not searched; aside from manually screening reference lists, no citation searching tools were used; no additional search methods such as PubMed Related Articles, personal reference libraries, or other database-embedded related-article recommendation features were employed; and stakeholders or content experts were not contacted to identify additional studies, as the designed search was considered sufficiently comprehensive through database coverage alone. Although corresponding authors were contacted via email regarding missing or ambiguous data during the data extraction process, no authors, experts, manufacturers, or other parties were specifically contacted to identify additional studies or unpublished data for inclusion in this review. The search strategy did not undergo formal external peer review (such as the PRESS checklist process) but was cross-checked and finalized by investigators within the research team. A complete PRISMA-S checklist is provided in Checklist 1.

### Selection Process

EndNote X9.3.3 (Clarivate Analytics, US) was used for reference management and automated deduplication, followed by manual verification. Two reviewers (JL and HT) independently screened titles and abstracts, then full texts against eligibility criteria. Discrepancies were resolved by discussion, with arbitration by a third reviewer (QF). Interrater agreement was assessed using the Kappa statistic.

### Data Collection Process

Two reviewers (JL and WX) independently extracted data using a predesigned form (WPS Office Excel). Extracted items included: title, first author, year, study design, LLM model, model modality, application scenario, prompt engineering approach, input/output formats, and outcome measures. Interreviewer consistency was calculated; disagreements were resolved by a third reviewer (QF). For missing or ambiguous data, corresponding authors were contacted via email; if unavailable after 2 weeks, items were recorded as “not reported” and excluded from descriptive analyses. No imputation was applied. For studies reporting multiple outcomes, we gave preference to the primary outcome defined by the authors; if none was specified, we selected the metric most central to the study’s objective through consensus between 2 reviewers. For other types of outcomes, we extracted the reported values without modification.

### Data Items

To manage the inherent overlap between technical tasks, studies were categorized based on their primary terminal clinical objective. For instance, studies employing information extraction specifically to enable automated TNM staging were classified under “Auxiliary Diagnosis” rather than “Information Extraction” to prioritize clinical utility over technical subprocesses.

### Study Risk of Bias Assessment

Following Omar and Levkovich [39], the included studies were classified and evaluated based on the assessment design and outcome indicators of the studies rather than their clinical application fields. QUADAS-2 (Quality Assessment of Diagnostic Accuracy Studies-2) [40] was applied for diagnostic accuracy studies validating LLM performance against histopathological diagnosis, endoscopist consensus, or clinical guidelines. PROBAST (prediction model risk of bias assessment tool) [41] was applied for prediction model studies focusing on the development and validation of LLM-based predictive models. ROBINS-I (Risk of Bias in Nonrandomized Studies - of Interventions) [42] was applied for nonrandomized intervention studies evaluating the LLM application effect or clinical value, including information extraction and knowledge-based tasks. Study classifications and corresponding tools are detailed in Multimedia Appendix 2.

Given that LLM studies differ from conventional clinical trials, 2 oncology experts (QF) made minor framework-preserving adaptations to each tool; specific adaptations are documented in Multimedia Appendix 3. Assessment was conducted independently by 2 researchers (JL and HT), with a third (WX) resolving disagreements. Final results were reviewed by an expert (QF). Interrater agreement was evaluated using the Kappa statistic. Overall evidence strength was evaluated considering study quality, consistency of findings, and methodological limitations.

### Synthesis Methods

Given the anticipated heterogeneity in clinical tasks, study designs, and outcome constructs, narrative synthesis following SWiM reporting guidelines [38] was planned a priori rather than quantitative meta-analysis. Meta-analysis was not conducted for four reasons: (1) substantial heterogeneity across fundamentally different clinical tasks, rendering pooled estimates uninterpretable; (2) a high proportion of studies rated at moderate, serious, or high risk of bias; (3) fewer than 5 studies within any subgroup sharing comparable task definitions, input modalities, and reference standards; and (4) marked inconsistency in outcome measures precluding standardized effect size extraction.

### Reporting Bias Assessment

This systematic review employed a narrative synthesis and did not perform statistical tests for publication bias. Given the absence of a quantitative meta-analysis and the substantial heterogeneity in study design and outcome reporting across included studies, methods such as funnel plots were considered inapplicable. During evidence synthesis and result interpretation, the research team conducted a qualitative assessment of potential reporting bias. By comparing the consistency between study objectives, methods, and reported outcomes, and by incorporating study registration information (where available) and author explanations, the team cautiously discussed the potential impact of missing results on study conclusions.

## Results

### Study Selection

**Figure 1.**
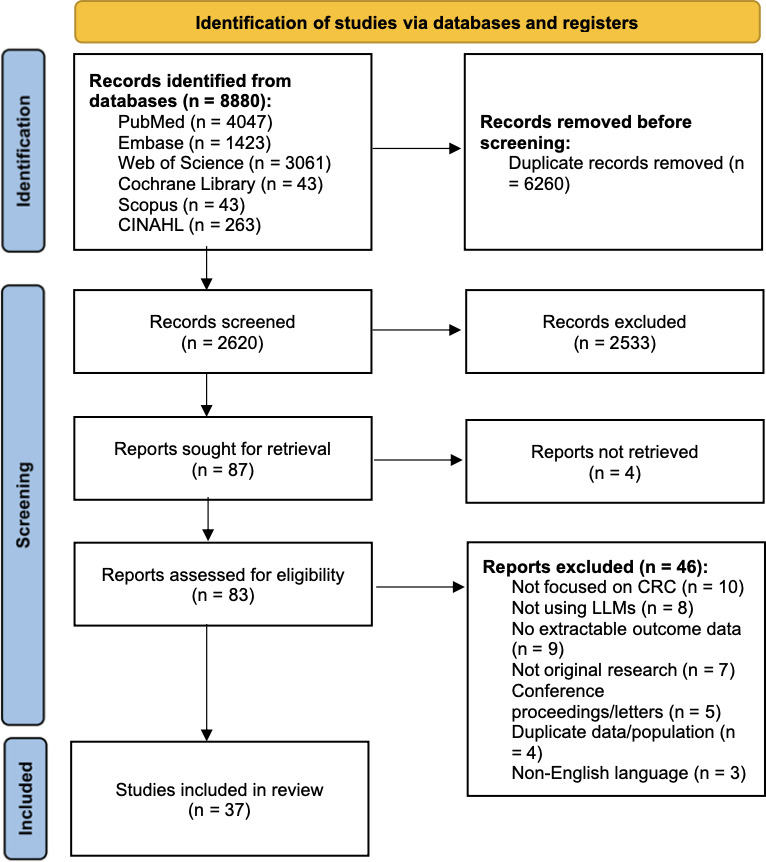
PRISMA (Preferred Reporting Items for Systematic Reviews and Meta-Analyses) 2020 flow diagram of the study selection process for examining the role of LLMs in colorectal cancer. CRC: colorectal cancer; LLM: large language model.

A total of 8880 records were retrieved (PubMed: 4047; Embase: 1423; Web of Science: 3061; Cochrane Library: 43; Scopus: 43; CINAHL: 263). After automated and manual deduplication using EndNote X9.3.3, 6260 unique records were identified. Following title/abstract screening, 2533 full-text articles were assessed, and 37 studies met the inclusion criteria. The screening-stage Kappa was 0.85. The screening process is presented in Figure 1.

### Study Characteristics

The data extraction consistency rate was 0.97. All 37 studies were published between 2023 and 2026, 2 in 2023 [22,43], 11 in 2024 [7,14,25,28,44-50], 22 in 2025 [8,10,13,17,51-67], and 2 in 2026 [20,68]. Studies primarily originated from China [7,20,25,49,50,54-57,59,64,65] and the United States [13,14,17,45,47,53,58,60,66,68], with others from Italy [62,63], Germany [46,61], Singapore [28,51], Israel [43], Switzerland [67], Spain [52], Turkey [48], the United Kingdom [44], South Korea [69], and multinational collaborations [8,10,22]. Application domains included auxiliary diagnosis [14,17,44,46,49,50,62,65], information extraction [10,17,44,52,57,69], knowledge-based question answering [7,22,25,28,45,48,50,63,64], treatment decision-making [8,20,47,53,56,61,67,68], predictive modeling [51,54], scientific research [58,66], and aided nursing [60].

The LLMs used varied widely, with the most frequent being OpenAI’s GPT series. Other models included Google’s Gemini, Anthropic’s Claude, Meta’s LLaMA series, as well as DeepSeek, GLM, and Qwen, among others. The best-performing models identified in comparative studies are summarized in Table 2. The results suggest that models such as GPT-4, GPT-4o, and Claude 2.1 showed relatively favorable performance in some tasks [14,25,28,68,69]; o3-mini reportedly showed comparatively higher intra-model stability and expert concordance among reasoning-oriented models for multidisciplinary team decision simulation [20]. However, for specific tasks, lightweight models or domain-specialized models may also perform optimally [17,51,52]. A summary of these details is provided in Table 2.

**Table 2.** Summary of included sources. Pure LLM: text-only language model processing textual inputs exclusively. Multimodal VLM: vision-language model capable of processing both textual and visual inputs (eg, GPT-4V, GPT-4o with image input).

Study	Country	LLMs^a^ used	Model type	Application domain	Best performer
Zeng, 2025 [8]	Multi-national	ChatGPT-4.5	Pure LLM	Treatment Decision	—^b^
Zeng, 2025 [56]	China	ChatGPT-4o, DeepSeek	Pure LLM	Treatment Decision	—
Schmutz, 2025 [61]	Germany	ChatGPT 4.0	Pure LLM	Treatment Decision	ChatGPT 4.0
Chatziisaak, 2025 [67]	Switzerland	ChatGPT-4	Pure LLM	Treatment Decision	—
Horesh, 2025 [53]	United States	ChatGP -3.5	Pure LLM	Treatment Decision	—
Kaiser, 2024 [47]	United States	ChatGPT-3.5, Microsoft Copilot	Pure LLM	Treatment Decision	—
Garg, 2026 [68]	United States	ChatGPT-4o	Pure LLM	Treatment Decision	GPT-4o
Qu, 2026 [20]	China	ChatGPT-o3-mini, DeepSeek-R1, Qwen qwq-plus	Pure LLM	Treatment Decision	o3-mini
Diaz, 2025 [66]	United States	AI-HOPE (LLaMA 3-based)	Pure LLM	Scientific Research	—
Yang, 2025 [58]	United States	LLaMA 3	Pure LLM	Scientific Research	—
Yang, 2025 [54]	China	BGE-M3, XGBoost	Pure LLM	Predictive Modeling	XGBoost
Kim, 2025 [51]	Singapore	BioBERT-Large, RadImageNet, 3D ResNet	Multimodal VLM	Predictive Modeling	BioBERT-Large
Lim, 2024 [28]	Singapore	GPT-4	Pure LLM	Knowledge QA	GPT-4
Hu, 2025 [64]	China	ChatGPT-4.5	Pure LLM	Knowledge QA	ChatGPT-4.5
Peng, 2024 [7]	China	ChatGPT-3.5	Pure LLM	Knowledge QA	—
Wang, 2024 [50]	China	GPT-3.5-turbo	Pure LLM	Knowledge QA	—
Zhang, 2025 [55]	China	ChatGPT-4o, Claude 3.5, DeepSeek	Pure LLM	Knowledge QA	ChatGPT-4o
Zhou, 2024 [25]	China	ChatGPT, Doctor GPT, Llama-2-70B, Mixtral-8 × 7B, Bard, Claude 2.1	Pure LLM	Knowledge QA	Claude 2.1
Gorelik, 2023 [43]	Israel	ChatGPT-4	Pure LLM	Knowledge QA	—
Maida, 2025 [63]	Italy	ChatGPT-4o	Pure LLM	Knowledge QA	—
Maida, 2025 [10]	Multi-national	ChatGPT-4	Pure LLM	Knowledge QA	—
Emile, 2023 [22]	Multi-national	ChatGPT-3.5	Pure LLM	Knowledge QA	—
Kepez, 2024 [48]	Turkey	ChatGPT-4	Pure LLM	Knowledge QA	ChatGPT-4
Atarere, 2024 [45]	United States	ChatGPT, BingChat, YouChat	Pure LLM	Knowledge QA	ChatGPT, YouChat
Yu, 2025 [57]	China	Gemini, GPT-4, GPT-4o, Claude, Llama, DeepSeek, GLM, Qwen	Pure LLM	Information Extraction	GPT-4
Chizhikova, 2025 [52]	Spain	RoBERTa	Pure LLM	Information Extraction	Task-specific models
Alzaid, 2024 [44]	UK	ChatGPT-4 Turbo, GPT-4V	Multimodal VLM	Information Extraction	—
Johnson, 2025 [17]	United States	Gemma-2-9B-It-SPPO, Llama-3-8B-Instruct	Pure LLM	Information Extraction	Gemma-2
Kim, 2025 [69]	South Korea	GPT-4	Pure LLM	Information Extraction	GPT-4
Ding, 2025 [65]	China	ChatGPT-4	Multimodal VLM	Auxiliary Diagnosis	—
Liu, 2024 [49]	China	ChatGPT-3.5, ChatGPT-4.0	Pure LLM	Auxiliary Diagnosis	GPT-4.0
Wang, 2025 [59]	China	ChatGPT, Claude, ERNie, SAM	Multimodal VLM	Auxiliary Diagnosis	—
Ferber, 2024 [46]	Germany	ChatGPT-4V	Multimodal VLM	Auxiliary Diagnosis	GPT-4V
Massimi, 2025 [62]	Italy	ChatGPT-4o	Multimodal VLM	Auxiliary Diagnosis	GPT-4o
Amini, 2025 [13]	United States	GPT-3.5-turbo, Bard (PaLM 2)	Pure LLM	Auxiliary Diagnosis	ChatGPT-3.5
Chang, 2024 [14]	United States	ChatGPT-4	Pure LLM	Auxiliary Diagnosis	ChatGPT-4
Sehgal, 2025 [60]	United States	ChatGPT-4.1	Pure LLM	Aided Nursing	—

a LLM: large language model.

b No intermodel comparison was performed or the metric is not applicable.

### Prompt Engineering and Model Training

The data extraction consistency rate was 0.97. We synthesized prompt engineering strategies, model inputs/outputs, and evaluation metrics (Table 3). Five studies [10,22,45,47,63] did not explicitly describe prompting strategies, employing basic queries primarily for educational purposes. Thirty-two studies described distinct methods, including instruction templates and instructional prompts [8,13,14,17,20,28,43,44,48,55,58-62,64-69], zero-shot learning [7,25,49,50,53,57,64], few-shot learning [46,56], fine-tuning [51,52,54], and hybrid approaches [57,60,68]. Training data were text-based in 33 studies [7,8,10,13,14,17,20,22,25,28,43-45,47-50,52-61,63,64,66-69], image-based in 2 studies [46,62], and multimodal in 2 studies [51,65]. Common outcome metrics included accuracy [7,8,17,20,25,28,44,46,48-50,52,53,55-57,59,60,62,64,65,68], *F*_1_-score [17,51,52,57,65], area under the curve [51,54,59], sensitivity [53,57,65], and concordance rate [13,14,17,20,28,43,44,48,56,59-61,68]. A categorized summary is provided in Multimedia Appendix 4.

**Table 3.** Prompt engineering and model training.

Study	Prompt method or content	Model input	Model output	Outcome indicators
Zeng, 2025 [8]	Instruction template	Standardized patient cases	Screening and monitoring recommendations	Correct/partially correct/incorrect proportions; descriptive statistics
Amini, 2025 [13]	Instruction template	Colonoscopy reports, pathology, history, family history	Colonoscopy interval recommendation	Agreement percentage, Fleiss’ kappa, McNemar test
Chang, 2024 [14]	Instruction template	Deidentified clinical data, colonoscopy reports, pathology reports	Follow-up colonoscopy interval suggestions	Agreement rate, Fleiss kappa
Johnson, 2025 [17]	Instruction template	Pathology report text	Yes/no answer	*F*_1_-score, PPV^a^, NPV^b^, sensitivity, specificity, MCC^c^
Lim, 2024 [28]	Instruction template	Patient scenario descriptions	Colonoscopy interval recommendations	Correct interval percentage, hallucination rate
Gorelik, 2023 [43]	Instruction template	Structured endoscopy reports & free-text clinical notes	Guideline-based next-step recommendations; Patient result explanation letters	Guideline adherence, accuracy, Fleiss’ kappa
Alzaid, 2024 [44]	Instruction template	Unstructured pathology reports	Structured JSON report with confidence	Accuracy, Kappa, AUROC^d^
Kepez, 2024 [48]	Instruction template	20 common questions on colon cancer	Answer text for each question	DISCERN, GQS, JAMA criteria, Flesch-Kincaid readability, SAM, HITS, VPI, HONcode
Zhang, 2025 [55]	Instruction template	Chinese Society of Clinical Oncology guideline standards / instructions	Colorectal cancer screening educational text	Accuracy, clarity, rigor scores
Yang, 2025 [58]	Instruction template	Natural language queries on clinical genomic data	Mutation profiles, survival curves, odds ratios	*P* values, hazard ratios, odds ratios
Wang, 2025 [59]	Instruction template	Free-text colonoscopy reports	Report-level labels	Accuracy, average precision, dice similarity coefficient, AUC^e^
Sehgal, 2025 [60]	Instruction template	Self-reported demographics	AI-generated personalized messages or chatbot dialogues	Intent score change, Cohen *d*, *P* values, OR^f^, Flesch-Kincaid readability
Schmutz, 2025 [61]	Instruction template	Clinical patient summaries and pathology reports	Treatment/diagnostic recommendations	Recommendation type, information density, consistency, level of evidence, time efficiency
Massimi, 2025 [62]	Instruction template	Colonoscopy video frames	Paris classification	Accuracy, sensitivity, specificity, Fleiss’ kappa
Ding, 2025 [65]	Instruction template	Pathology images and text prompts	Tissue origin, lesion classification, diagnosis	Accuracy, sensitivity, specificity, PPV, NPV, *F*_1_-score, Kappa, ICC^g^
Diaz, 2025 [66]	Instruction template	Natural language queries for scanning and validating clinical genomic datasets	Survival analysis results, mutation frequency comparisons, statistical significance	*P* values, odds ratios, survival rates
Chatziisaak, 2025 [67]	Instruction template	Patient clinical data	Treatment recommendation	Consistency, chi-square test
Qu, 2026 [20]	Instruction template; Multi-role prompting	Structured variables and free-text summaries from clinical records	Four-category treatment classification code	Intra-model agreement; expert-model concordance, Cohen κ
Garg, 2026 [68]	Instruction template; Role prompting; Few-shot; Chain-of-thought; JSON schema enforcement	Colonoscopy reports, pathology reports, patient family history and preoperative diagnoses	Structured clinical entities and 2020 USMSTF-based surveillance interval recommendations; 2024 ACG/ASGE quality indicators	Case-level accuracy, Cohen κ; Fleiss’ κ; ADR, SSLDR, cecal intubation rate, bowel prep adequacy
Kim, 2025 [69]	Instruction template; Role prompting	Unstructured preoperative abdominal CT / rectal MRI reports	Lesion location and cTNM stage and reasoning	Lesion location accuracy
Kim, 2025 [51]	Fine-tuning	CT images and radiology report texts	Binary NAR score classification	AUC
Chizhikova, 2025 [52]	Fine-tuning	Spanish colon MRI report texts, numerical features, categorical features	TNM^h^ staging	Accuracy, macro *F*_1_-score, precision, recall
Yang, 2025 [54]	Fine-tuning	Clinical EHR^i^ data	Binary colorectal adenoma risk	AUC, sensitivity, specificity, *F*_1_-score, PPV, NPV, mean lead time
Ferber, 2024 [46]	Few-shot	Cancer pathology images	Image classification labels	Accuracy, confidence interval, recall
Zeng, 2025 [56]	Few-shot; Role prompting; Context learning	Real-world pathology report text	Recommendation on need for additional surgery	Accuracy; guideline consistency proportion
Peng, 2024 [7]	Zero-shot	Medical questions from books	Colorectal cancer-related answers	Accuracy, comprehensiveness scores
Zhou, 2024 [25]	Zero-shot	150 CRC-related^j^ closed-ended questions	Yes/no answers	Accuracy
Liu, 2024 [49]	Zero-shot	Colorectal cancer case report texts	Primary/secondary diagnoses	Accuracy
Wang, 2024 [50]	Zero-shot	Pathology report text and related questions	Answers to pathology questions	7-point Likert scale
Horesh, 2025 [53]	Zero-shot	Clinical patient summaries	Next best management recommendation	Consistency with multidisciplinary team decisions, reasonableness score, interrater reliability
Yu, 2025 [57]	Zero-shot; Chain-of-thought	Endoscopy/colonoscopy report texts	Structured JSON including lesion location, features, layer structure, distribution, diagnosis	Precision, recall, *F*_1_-score, accuracy
Hu, 2025 [64]	Zero-shot	Patient question texts	Answer texts	Accuracy, completeness, clarity scores
Maida, 2025 [10]	*—* ^k^	15 questions on colorectal cancer screening	Text answers to questions	Accuracy, completeness, clarity scores
Emile, 2023 [22]	*—*	38 common questions on CRC prevention, diagnosis, management	Text answers	Expert consensus; consistency with guidelines
Atarere, 2024 [45]	*—*	15 questions on CRC screening concepts and 5 experience-based questions	Response appropriateness	Appropriateness rating
Kaiser, 2024 [47]	*—*	Clinical scenario questions on next management	Text recommendations for clinical questions	Accuracy score, consistency, verbosity
Maida, 2025 [63]	*—*	Patient queries	ChatGPT-generated answers	Expert scores, patient scores

a PPV: positive predictive value.

b NPV: negative predictive value.

c MCC: Matthews correlation coefficient.

d AUROC: area under the receiver operating characteristic curve.

e AUC: area under the curve.

f OR: odds ratio.

g ICC: intraclass correlation coefficient.

h TNM: tumor–node–metastasis.

i EHR: electronic health record.

j CRC: colorectal cancer.

k Prompt method was not explicitly reported.

### Risk of Bias in Studies

**Figure 2.**
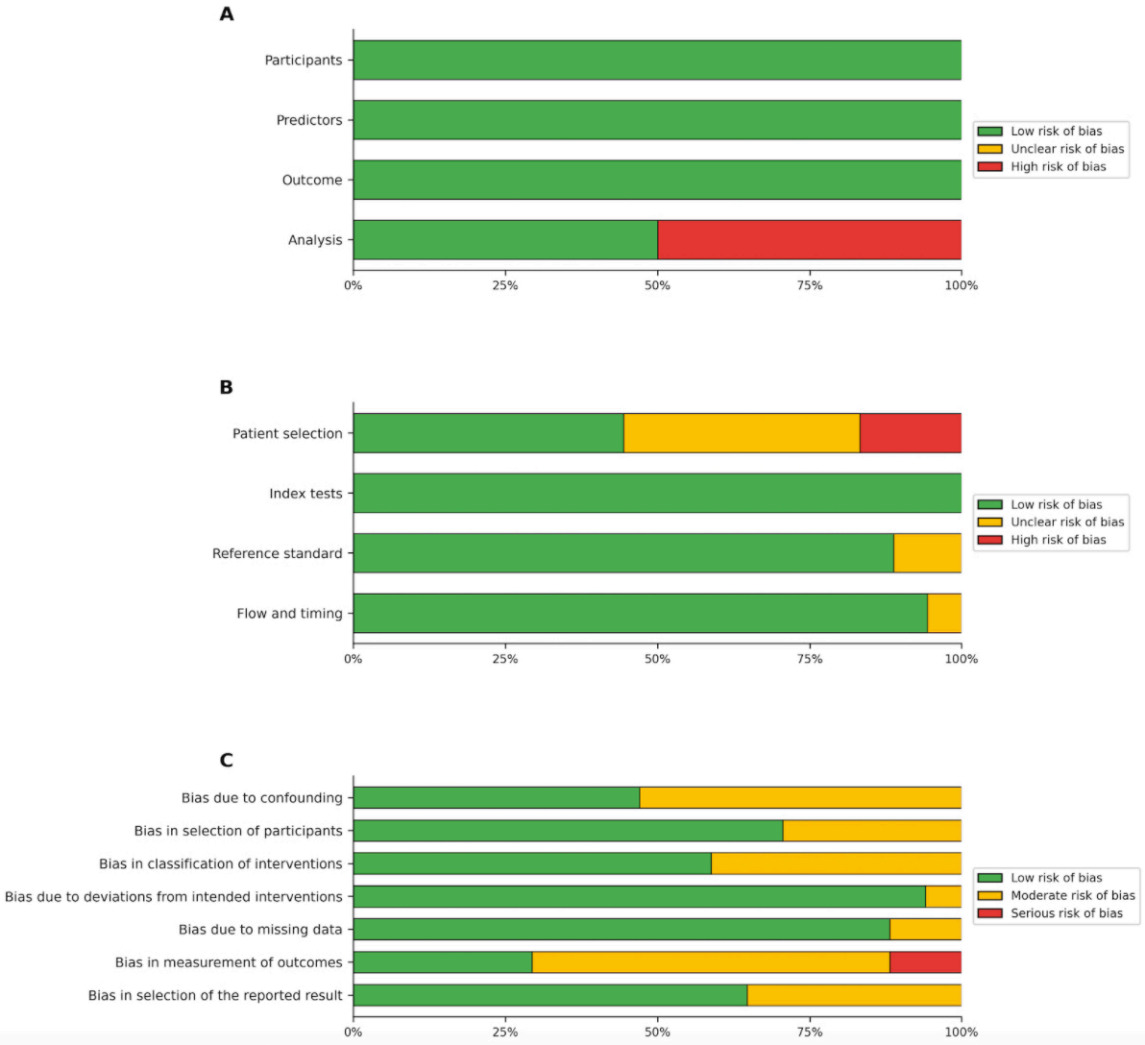
(A) The quality appraisal for 2 predictive studies with PROBAST (prediction model risk of bias assessment tool). (B) The quality appraisal for 18 diagnostic studies with QUADAS-2 (Quality Assessment of Diagnostic Accuracy Studies-2). (C) The quality appraisal for 17 intervention trials with ROBINS-I (Risk of Bias in Nonrandomized Studies - of Interventions).

The included studies were categorized by research objective, and quality was assessed using the corresponding appraisal tool. The kappa value between the 2 reviewers was 0.95. Two predictive modeling studies [51,54] were evaluated using PROBAST (Figure 2A); both showed low risk of bias across the participants, predictors, and outcome domains, but one exhibited high risk of bias in the analysis domain. Eighteen diagnostic studies [13,14,17,25,28,43-46,49,52,56,59,62,65,67,68] were assessed using QUADAS-2 (Figure 2B); while most demonstrated acceptable applicability, risk of bias in the patient selection domain was frequently unclear or high. Seventeen intervention studies [7,8,10,20,22,47,48,53,55,58,60,61,63,64,66,69] were appraised using ROBINS-I (Figure 2C); risk of bias was predominantly low for participant selection, deviations from intended interventions, and missing data, but moderate to serious for outcome measurement and classification of interventions.

Overall, 27 of the 37 included studies were rated above low risk of bias: 6 as high or serious [28,44-46,57,59], 7 as unclear [43,47,50,52,53,62,65], and 14 as moderate [7,8,10,13,20,22,48,55,58,60,61,63,64,66], while 10 were rated as low [14,17,25,49,51,54,56,67-69]. The most problematic domains across tools were outcome measurement [8,10,13,20,22,43,46-48,50,52,53,55,58,60-62,64,66,68] and patient selection [25,28,44-46,52,56,59,62,65]. Given these recurring concerns, particularly regarding blinding [43,46,53,60,61], outcome measurement [8,10,13,20,22,43,46-48,50,52,53,55,58,60-62,64,66,68], and confounding [17,20,44-46,53,58,60-62,66,68], and the considerable heterogeneity in clinical tasks, LLM models, and outcome metrics, the overall certainty of evidence was judged as moderate to low. Quantitative meta-analysis was not feasible; even within the largest subgroup, fewer than 5 studies were sufficiently aligned in task definition, input modality, and reference standard to permit reliable pooling. A narrative synthesis was therefore adopted, and the findings should be interpreted with caution.

## Discussion

### Principal Findings

Through a comprehensive analysis of 37 studies, we identified 5 primary application domains of LLMs in CRC diagnosis and treatment: auxiliary diagnosis, information extraction, knowledge-based question-answering and patient education, treatment decision support, and scientific research and predictive modeling (Table 2). These domains are often interconnected in clinical practice. For instance, information extraction frequently provides structured data to support diagnostic processes [17,57], while knowledge-based question-answering is widely applied in scientific communication and patient education [7,45,48,63,64].

### Applications of LLMs in CRC

LLMs enable the automated extraction of clinical features through NLP [70]. Multiple studies have utilized LLMs to extract key information from EHRs [17], endoscopy reports [27], radiology reports [25], and pathology reports [17,52]. This capability assists not only in clinical staging and histological classification [71] but also in predicting disease progression and treatment response [17]. For instance, lymph node metastasis assessment based on MRI reports [51] and tumor progression prediction from radiology reports [72] have shown promising accuracy. These advancements underscore the significant value of LLMs in early CRC screening. Early diagnosis can effectively improve survival rates [25], and mass screening achieves a high detection rate for early-stage lesions [73]. Wang leveraged LLMs to automatically extract knowledge from colonoscopy image-text records, enabling polyp detection and segmentation without manual annotation, thereby offering a novel approach to screening automation [59]. A systematic review of LLMs in gastroenterology similarly demonstrated the potential applications of LLMs in gastrointestinal endoscopy and precancerous lesion screening [74]. Despite challenges such as insufficient extraction performance for complex tasks and hallucinations reporting a lower accuracy of 55% for LLMs in classifying pedunculated polyps, indicating they cannot yet fully replace endoscopic experts [25,62], we remain optimistic about their future performance in assisting CRC diagnosis and early screening. This optimism is fueled by ongoing advancements in multimodal integration [75], the development of domain-specific models [46,76], and the continuous optimization of training data [34].

Leveraging their strong interactive capabilities and extensive knowledge, LLMs are widely evaluated for CRC medical question-answering and patient education [7,22]. Furthermore, advancing multimodal models now enable LLMs to jointly analyze medical images and text, offering CRC diagnostic and therapeutic suggestions in controlled settings [6]. Gong has recently emphasized that multimodal fusion has emerged as the dominant next-generation development trend for gastrointestinal artificial intelligence [9]; however, this important technological milestone has not yet received adequate attention in available systematic reviews. Ferber demonstrated that multimodal LLMs applying in-context learning achieved near-pathologist-level classification of cancer pathology images [46], and Kim [51] showed that combined LLM and vision deep learning architectures outperformed either modality alone for neoadjuvant rectal score prediction, which preliminarily suggests the potential of multimodal LLMs, as they can reach a level close to that of pathologists when processing pathological image classification and clinical prediction tasks, and outperform single-modality models. Despite this progress, the diagnostic accuracy of current multimodal models on morphologically complex tasks remains constrained [34,62]. This reinforces the prevailing clinical consensus that current LLMs must be deployed strictly as decision-support adjuncts rather than autonomous diagnostic agents, thereby mitigating the significant clinical risks associated with automation bias and diagnostic delay [23,49]. Furthermore, extraction performance varied markedly, dictated by underlying model architecture and optimization strategy. GPT-4, augmented with multi-strategy prompting, appeared to outperform zero-shot baselines for colonoscopy report extraction [57], while biomedical pretrained RoBERTa showed better performance than general-purpose GPT models for TNM staging in the available evidence from Spanish-language reports [52]. This discrepancy unequivocally indicates that domain-adaptive and language-specific pretraining confers fundamental structural semantic advantages that advanced prompt engineering alone cannot replicate [44], consistent with recent evaluations where specialized models exhibited superior performance within data-constrained clinical settings [77]. Nonetheless, the majority of this evidence is derived from retrospective analyses and single-center validations, with a notable paucity of prospective, multicenter clinical trials to confirm generalizability and real-world efficacy [47,55,66].

The NLP and named entity recognition capabilities of LLM extend their utility beyond direct clinical support for practitioners and patients, substantially improving the efficacy of medical research workflows [78]. In the domain of data extraction and analysis, Johnson leveraged the Gemma-2 model to accurately identify and extract key pathological diagnostic entities—such as dysplasia, high-grade dysplasia/adenocarcinoma, and invasive carcinoma—from unstructured pathology reports [17]. This high accuracy aligns robustly with Chen et al [6], who demonstrated comparable reliability in extracting oncological variables from EHRs, confirming automated information extraction as one of the most mature LLM applications. Beyond data retrieval, LLMs are increasingly serving as active engines for hypothesis generation [79,80]. Their probabilistic structure allows them to synthesize vast, disparate datasets and infer latent correlations that traditional algorithms might overlook [72]. In translational medicine, Yang developed AI-HOPE-TP53, a LLaMA 3-based conversational agent that facilitates pathway-centric analysis of clinical genomic data in early-onset CRC [58]. By rapidly generating statistical outputs like survival curves and hazard ratios, this system accelerates hypothesis-driven research in precision oncology [81]. The viability of this paradigm shift is further corroborated by Abdel-Rehim, who experimentally validated that LLM-driven pipelines can successfully identify novel, laboratory-verifiable synergistic drug combinations [80]. Furthermore, hybrid LLM architectures are democratizing access to complex analytical tools in routine practice. Yang et al [54] developed an early-stage CRC adenoma risk prediction model combining BGE-M3 semantic vector encoding with XGBoost algorithms. By enabling clinicians without specialized computational expertise to perform sophisticated risk stratification based on LLM-processed outputs, such models substantially reduce the administrative burden and facilitate a more patient-centered clinical workflow [6]. The research-supportive functions of LLMs have also expanded into foundational scholarly activities, including knowledge synthesis and the drafting of study protocols, ethics materials, and preliminary manuscript sections [43,59]. However, the originality and factual accuracy of such artificial intelligence-generated scholarly content necessitate rigorous human oversight to ensure scientific integrity [82].

### Limitations of LLMs and Future Directions

Current research on LLMs in the field of CRC predominantly focuses on textual data processing [83]; investigations into other modalities, including CT images [51,67], histopathological slides [58], and bioinformatics data [58], remain in their nascent stages, demonstrating suboptimal output precision and task stability. General-purpose LLMs (eg, ChatGPT and the LLaMA series), predominantly pretrained on public databases, frequently manifest deficiencies such as delayed knowledge base updates, insufficient coverage of CRC subspecialty knowledge, a propensity for hallucinations, and an absence of authoritative evidence-based support for pivotal clinical content [8,76,84]. Conversely, although existing medical-domain-specific LLMs (eg, Med-PaLM 2, BioBERT, and ClinicalBERT) possess certain advantages in general medical tasks, their comprehensive performance in complex subspecialty tasks, such as the precision treatment of CRC, still lags behind that of large-parameter general-purpose models [57]. More critically, the reliability and generalizability of currently well-developed diagnostic and decision-support tools are severely hindered by methodological flaws; existing evidence relies disproportionately on retrospective, single-center datasets lacking temporal or geographic stratification [77]. This evaluative paradigm renders models highly susceptible to overfitting and training data leakage, thereby precipitating a drastic degradation in performance within real-world clinical environments [85]. There remains a critical paucity of rigorous prospective, multicenter clinical validation data within this domain [86,87].

The risk-of-bias assessment revealed several recurring methodological weaknesses across study designs. Among diagnostic accuracy studies evaluated with QUADAS-2, the patient selection domain was the most common source of concern, with ratings of “unclear” or “high” largely attributable to unreported sampling procedures and potentially inappropriate exclusion criteria [32,34,39,42,57]. For nonrandomized intervention studies assessed with ROBINS-I, the principal limitations were inadequate adjustment for confounding variables [40,46-48,52,58,61,62] and the absence of blinded outcome assessment, both of which may bias effect estimates [15,19,37,41,44,47,51,52,54,55,58,62]. Prediction model studies appraised with PROBAST generally performed well in the participants, predictors, and outcome domains but showed weaknesses in the analysis domain, including limited sample size, unexplained participant attrition, and insufficiently described handling of missing data [43,45]. Collectively, these methodological limitations reduce the reliability of the current evidence base and constrain its translational applicability.

Beyond data-related constraints, the intrinsic technical vulnerabilities and compliance risks of LLMs pose substantial threats to clinical safety [23]. The profound sensitivity of models to version iterations and prompt variations results in exceedingly poor reproducibility of outputs across multi-institutional settings [77]. In the absence of specific instructional constraints, models are not only prone to hallucinations but may also exacerbate negative societal biases and stereotypes [88]. Uncritical acceptance of these recommendations by clinicians may engender bias, subsequently precipitating critical diagnostic delays or inappropriate clinical interventions [89]. Furthermore, constrained by the heterogeneity of patient requirements and the stringent governance of sensitive data, applications pertaining to patient follow-up and supportive care remain the most underdeveloped [60]. Moreover, the pervasive absence of data privacy and information security protocols during the cloud-based deployment of open-source LLMs further impedes their clinical translation and real-world implementation [6,9].

To address current technical bottlenecks, it is imperative to enhance model precision and reliability through future technological advancements [90]. Multimodal integration is recognized as the predominant trajectory for next-generation technological development in this domain, offering the potential to transcend the limitations of unimodal text processing [91]. Regarding optimization strategies, RAG technology emerges as an optimal solution for tailoring general-purpose models to subspecialty clinical scenarios [9]. By interfacing with independent, verifiable, and authoritative subspecialty knowledge bases, RAG facilitates real-time knowledge updates, effectively enhances the concordance between model outputs and authoritative guidelines, substantially mitigates hallucinations, and endows models with robust interpretability [92,93]. Concurrently, prompt engineering (eg, instruction templates, few-shot learning, and chain-of-thought prompting) can rapidly augment the performance of general-purpose models in specific tasks, including pathological data extraction, treatment regimen recommendation, and follow-up protocol formulation, without altering underlying model weights [94,95].

Regarding clinical integration and ethical governance, future research priorities must pivot toward achieving real-world validity and safety [96]. Primarily, prospective, multicenter clinical validations must be conducted for diagnostic and treatment planning applications, while patient follow-up and supportive care systems must be specifically developed to rectify deficiencies in full-cycle management [97]. More crucially, LLMs cannot supplant medical professionals; their responsible clinical application must be strictly predicated on the establishment of a robust ethical governance framework [98]. This necessitates the strict enforcement of their adjunctive role under continuous human supervision, concurrent with the resolution of data privacy issues and the assurance of foundational data quality [99]. Ultimately, cross-disciplinary collaboration is imperative to delineate accountability, ensuring the synchronous evolution of governance frameworks and cutting-edge technologies [82].

### Limitations of This Systematic Review

This review has several limitations. First, the majority of included studies were retrospective and single-center in design, and no prospective multicenter clinical trials establishing real-world LLM effectiveness in CRC care were identified. Only a minority conducted independent external validation, precluding confirmation of generalizability across diverse populations and institutions. Second, the rapid publication pace of LLM research means some recent developments may not have been captured despite the April 1, 2026 search cutoff. Third, restriction to English-language publications may introduce geographic bias. Fourth, several included studies evaluated proprietary commercial models such as GPT-4 and Claude, whose architectures and training data are not fully disclosed, introducing additional transparency and reproducibility concerns. No included study reported direct industry sponsorship for LLM evaluation. Finally, the search strategy was only cross-checked internally without formal external peer review, potentially leading to omission of a few unpublished or noncore journal studies. Inherent subjectivity in quality appraisal was mitigated through independent dual assessment, third-reviewer arbitration, and expert validation [100,101].

### Conclusions

This review establishes an integrative framework that synthesizes evidence across diverse study designs and LLM categories to compare their respective strengths and limitations in CRC care. Distinct from prior reviews that have addressed gastroenterology broadly or have been confined to a single study design, our work focuses specifically on the full-cycle CRC care continuum and, for the first time, comparatively evaluates general-purpose, domain-specific, and multimodal LLMs, thereby elucidating how prompt engineering and heterogeneous evaluation metrics shape reported outcomes. While our findings substantiate the clinical potential of LLMs, these results should be interpreted with caution, given the overall low quality of the available evidence. Most included studies failed to report key safeguards against bias—such as blinding of outcome assessors, adequate adjustment for confounders, or the use of prospective, multicenter designs to validate model generalizability. Moreover, the substantial heterogeneity we observed across task types, LLM categories, prompt engineering strategies, reference standards, and outcome measures indicates that the performance advantages reported for any specific LLM are confined to the corresponding tasks and clinical scenarios and cannot be generalized. Future efforts should therefore prioritize the integration of LLMs into real-world clinical practice, which will require prospective, multicenter validation, a robust privacy-protection framework, and rigorous human oversight to mitigate bias. Against the backdrop of a rising global CRC burden and persistent disparities in health care resource allocation, this review provides an evidence base to inform the clinical translation, equitable scaling, and policy formulation surrounding LLM deployment in CRC care.

### Registration and Protocol

This systematic review was prospectively registered in the International Prospective Register of Systematic Reviews (PROSPERO) under registration number CRD420251248261. The review protocol is publicly accessible through the PROSPERO database. No separate protocol manuscript was published.

One amendment was made to the registered protocol: the literature search cutoff date was extended from November 1, 2025 to April 1, 2026, to capture the most recent publications prior to data synthesis. This amendment was implemented after the initial search had been completed and did not alter the review’s eligibility criteria, synthesis methodology, or any other prespecified procedures. The narrative synthesis approach (SWiM), quality assessment tools (QUADAS-2, PROBAST, ROBINS-I), eligibility criteria, database selection, screening processes, and data extraction methods were all carried out as prespecified in the registered protocol. No other amendments were made.

## Supplementary material

10.2196/89862 Multimedia Appendix 1 Detailed literature search strategies.

10.2196/89862 Multimedia Appendix 2 Methodological classification, appraisal tools, and evaluation metrics of the included studies.

10.2196/89862 Multimedia Appendix 3 Documentation of framework-preserving adaptations to quality appraisal tools.

10.2196/89862 Multimedia Appendix 4 Prompt engineering strategies and application scenarios in the included studies.

10.2196/89862 Checklist 1 PRISMA‐S checklist.

## Abbreviations

CRC: colorectal cancer
EHR: electronic health record
LLM: large language model
NLP: natural language processing
PICOS: Population, Intervention, Comparison, Outcome, Study design
PRISMA: Preferred Reporting Items for Systematic Reviews and Meta-Analyses
PRISMA-S: Preferred Reporting Items for Systematic Reviews and Meta-Analyses Literature Search Extension
PROBAST: prediction model risk of bias assessment tool
PROSPERO: prospective register of systematic reviews
QUADAS-2: Quality Assessment of Diagnostic Accuracy Studies-2
RAG: retrieval-augmented generation
ROBINS-I: Risk of Bias in Nonrandomized Studies - of Interventions
SWiM: synthesis without meta-analysis
TNM: tumor–node–metastasis
