# Supplementary material for: Large Language Models in Colorectal Cancer Care and Clinical Decision Support: Systematic Review
Source: J Med Internet Res. 2026 May 21;28:e89862. doi: 10.2196/89862 (PMC13193707; doi:10.2196/89862)
Supplement: Multimedia Appendix 1 [file jmir-v28-e89862-s001.pdf]

Supplementary Appendix 1: Detailed Literature Search Strategies

**1.Database: MEDLINE(through PubMed)**		
**Last searched:** March 1, 2026		
**Search ID**	**Query**	**Results**
#1	'colonic neoplasms'[MeSH Terms]	87090
#2	'Rectal Neoplasms'[MeSH Terms]	58757
#3	'colon cancer*'[tiab] OR 'colon neoplasm*'[tiab] OR 'colorectal cancer*'[tiab] OR 'colon tumor*'[tiab] OR 'colon carcinoma'[tiab] OR 'colon malignancy'[tiab] OR 'carcinoma of colon[tiab] AND rectum[tiab]' OR 'colorectal carcinoma'[tiab] OR 'colorectal malignanc*'[tiab] OR 'colorectal neoplasm*'[tiab] OR 'colorectal cancer*'[tiab] OR 'colorectal tumor*'[tiab] OR 'large bowel cancer*'[tiab] OR 'cancer* of the colon'[tiab] AND 'rectum'[tiab] OR 'colorectal adenocarcinoma'[tiab] OR 'rectal cancer*'[tiab] OR 'cancer* of the rectum'[tiab] OR 'colorectal cancer*'[tiab] OR 'carcinoma of the rectum'[tiab] OR 'rectal malignanc*'[tiab] OR 'rectal neoplasm*'[tiab] OR 'rectal tumor*'[tiab]	205911
#4	#1 OR #2 OR #3	293366
#5	'large language models'[MeSH Terms]	2034
#6	'artificial intelligence'[MeSH Terms]	277162
#7	'generative artificial intelligence'[MeSH Terms]	1717
#8	'language model*'[tiab] OR 'LLM'[tiab] OR 'AI'[tiab] OR 'generat* artific* intellig*'[tiab] OR 'GPT'[tiab] OR 'ChatGPT'[tiab] OR 'Claude'[tiab] OR 'Gemini'[tiab] OR 'LLaMA'[tiab] OR 'transform* model*'[tiab] OR 'foundat* model*'[tiab] OR 'Kimi'[tiab] OR 'DeepSeek'[tiab] OR 'Grok'[tiab] OR 'Qwen'[tiab] OR 'Doubao'[tiab] OR 'seed'[tiab] OR 'ERNIE'[tiab] OR 'Moonshot'[tiab] OR 'Mistral'[tiab]	256357
#9	#5 OR #6 OR #7 OR #8	494414
#10	#4 AND #9	4047
**2.Database: Embase**		
Last searched: March 1, 2026		
#1	'colon tumor'/exp	211674
#2	'rectum tumor'/exp	99054
#3	((((((((((((((((((colonic AND neoplasm* OR rectal) AND neoplasm* OR colon) AND cancer* OR colon) AND neoplasm* OR colorectal) AND cancer* OR colon) AND tumor* OR colon) AND carcinoma OR colon) AND malignancy OR (carcinoma AND of AND colon AND rectum) OR colorectal) AND carcinoma OR colorectal) AND malignanc* OR colorectal) AND neoplasm* OR colorectal) AND cancer* OR colorectal) AND tumor* OR large) AND bowel AND cancer* OR (cancer* AND of AND the AND colon AND rectum) OR colorectal) AND adenocarcinoma OR rectal) AND cancer* OR (cancer* AND of AND the AND rectum) OR colorectal) AND cancer*	47402

	OR (carcinoma AND of AND the AND rectum) OR rectal) AND malignanc* OR rectal) AND neoplasm* OR rectal) AND tumor*:ti,ab,kw	
#4	#1 OR #2 OR #3	304825
#5	'large language model'/exp	20254
#6	'artificial intelligence'/exp	196751
#7	'generative artificial intelligence'/exp	21108
#8	(((language AND model* OR llm OR ai OR generat*) AND artific* AND intellig* OR gpt OR chatgpt OR claude OR gemini OR llama OR transform*) AND model* OR foundat*) AND model* OR kimi OR deepseek OR grok OR qwen OR doubao OR seed OR ernie OR moonshot OR mistral:ti,ab,kw	862727
#9	#5 OR #6 OR #7 OR #8	1006508
#10	#4 AND #9	7611
#11	#10 AND (2022:py OR 2023:py OR 2024:py OR 2025:py OR 2026:py) AND 'human'/de AND 'article'/it	1423
**3.Database: Cochrane Library**		
**Last searched:** March 1, 2026		
**Search ID**	**Query**	**Results**
#1	colonic neoplasm* OR Rectal Neoplasm* OR colon cancer* OR colon neoplasm* OR colorectal cancer* OR colon tumor* OR colon carcinoma OR colon malignancy OR (carcinoma of colon and rectum) OR colorectal carcinoma OR colorectal malignanc* OR colorectal neoplasm* OR colorectal cancer* OR colorectal tumor* OR large bowel cancer* OR (cancer* of the colon and rectum) OR colorectal adenocarcinoma OR rectal cancer* OR (cancer* of the rectum) OR colorectal cancer* OR (carcinoma of the rectum) OR rectal malignanc* OR rectal neoplasm* OR rectal tumor* in Title Abstract Keyword - (Word variations have been searched)	162
#2	Large Language Models OR Artificial Intelligence OR Generative Artificial Intelligence OR language model* OR LLM OR AI OR generat* artific* intellig* OR GPT OR ChatGPT OR Claude OR Gemini OR LLaMA OR transform* model* OR foundat* model* OR Kimi OR DeepSeek OR Grok OR Qwen OR Doubao OR seed OR ERNIE OR Moonshot OR Mistral in Title Abstract Keyword - (Word variations have been searched)	2064
#3	#1 AND #2	43
**4.Database: Web of Science**		
**Last searched:** March 1, 2026		
**Search ID**	**Query**	**Results**
#1	'colonic neoplasm*' OR 'rectal neoplasm*' OR 'colon cancer*' OR 'colon neoplasm*' OR 'colorectal cancer*' OR 'colon tumor*' OR 'colon carcinoma' OR 'colon malignancy' OR 'colorectal carcinoma' OR 'colorectal malignanc*' OR 'colorectal neoplasm*' OR 'colorectal	336014

	tumor*' OR 'large bowel cancer*' OR 'colorectal adenocarcinoma' OR 'rectal cancer*' OR 'rectal malignanc*' OR 'rectal tumor*'	
#2	'language model*' OR 'LLM' OR 'artificial intelligence' OR 'generative artificial intelligence' OR 'GPT' OR 'ChatGPT' OR 'Claude' OR 'Gemini' OR 'LLaMA' OR 'transformer model*' OR 'foundation model*' OR 'Kimi' OR 'DeepSeek' OR 'Grok' OR 'Qwen' OR 'Doubao' OR 'ERNIE' OR 'Moonshot' OR 'Mistral'	457611
#3	#1 AND #2	3061
**5.Database: Scopus**		
**Last searched:** March 1, 2026		
**Search ID**	**Query**	**Results**
#1	'colonic neoplasm*' OR 'rectal neoplasm*' OR 'colon cancer*' OR 'colon neoplasm*' OR 'colorectal cancer*' OR 'colon tumor*' OR 'colon carcinoma' OR 'colon malignancy' OR 'colorectal carcinoma' OR 'colorectal malignanc*' OR 'colorectal neoplasm*' OR 'colorectal tumor*' OR 'large bowel cancer*' OR 'colorectal adenocarcinoma' OR 'rectal cancer*' OR 'rectal malignanc*' OR 'rectal tumor*'	24530
#2	'language model*' OR 'LLM' OR 'artificial intelligence' OR 'generative artificial intelligence' OR 'GPT' OR 'ChatGPT' OR 'Claude' OR 'Gemini' OR 'LLaMA' OR 'transformer model*' OR 'foundation model*' OR 'Kimi' OR 'DeepSeek' OR 'Grok' OR 'Qwen' OR 'Doubao' OR 'ERNIE' OR 'Moonshot' OR 'Mistral'	1095525
#3	#1 AND #2	43
**6.Database: CINAHL**		
**Last searched:** March 1, 2026		
**Search ID**	**Query**	**Results**
#1	SU('colonic neoplasm*' OR 'rectal neoplasm*' OR 'colon cancer*' OR 'colon neoplasm*' OR 'colorectal cancer*' OR 'colon tumor*' OR 'colon carcinoma' OR 'colon malignancy' OR 'colorectal carcinoma' OR 'colorectal malignanc*' OR 'colorectal neoplasm*' OR 'colorectal tumor*' OR 'large bowel cancer*' OR 'colorectal adenocarcinoma' OR 'rectal cancer*' OR 'rectal malignanc*' OR 'rectal tumor*')	56183
#2	SU('language model*' OR 'LLM' OR 'artificial intelligence' OR 'generative artificial intelligence' OR 'GPT' OR 'ChatGPT' OR 'Claude' OR 'Gemini' OR 'LLaMA' OR 'transformer model*' OR 'foundation model*' OR 'Kimi' OR 'DeepSeek' OR 'Grok' OR 'Qwen' OR 'Doubao' OR 'ERNIE' OR 'Moonshot' OR 'Mistral')	713743
#3	#1 AND #2	263
