# Supplementary material for: Large Language Models in Colorectal Cancer Care and Clinical Decision Support: Systematic Review
Source: J Med Internet Res. 2026 May 21;28:e89862. doi: 10.2196/89862 (PMC13193707; doi:10.2196/89862)
Supplement: Multimedia Appendix 2 [file jmir-v28-e89862-s002.pdf]

Multimedia Appendix 2. Methodological classification, appraisal tools, and evaluation metrics of the included studies.

Study	Original Application Domain	Appraisal Tool	Methodological Justification	Outcome Indicators
Yang, 2025	Predictive Modeling	PROBAST	Designed to forecast future adenoma risk based on historical patient EHR data, meeting criteria for multivariable prediction model development and validation.	AUC, sensitivity, specificity, F1-score, PPV, NPV, mean lead time.
Kim, 2025	Predictive Modeling	PROBAST	Utilizes multimodal models to predict future neoadjuvant rectal (NAR) scores, constituting a clinical-outcome prediction model.	AUC.
Ding, 2025	Auxiliary Diagnosis	QUADAS-2	Image classification outputs were compared against histopathological ground truth to calculate diagnostic accuracy.	Accuracy, sensitivity, specificity, PPV, NPV, F1-score, Kappa, ICC.
Liu, 2024	Auxiliary Diagnosis	QUADAS-2	Deterministic primary/secondary diagnoses from case-report texts were compared against clinical reference standards to calculate classification accuracy.	Accuracy.
Wang, 2025	Auxiliary Diagnosis	QUADAS-2	Extracted binary labels of polyp presence were compared against annotated ground truth; segmentation performance was evaluated with DSC and AUC.	Accuracy, AP, DSC, AUC.
Ferber, 2024	Auxiliary Diagnosis	QUADAS-2	Direct classification of cancer pathology images against expert-annotated labels represents a standard diagnostic index-test design.	Accuracy, confidence interval, recall.
Massimi, 2025	Auxiliary Diagnosis	QUADAS-2	Interpretation of Paris polyp classification from colonoscopy video frames was benchmarked against human expert gold	Accuracy, sensitivity, specificity, Fleiss' kappa.

			standards.	
Amini, 2025	Auxiliary Diagnosis	QUADAS-2	Colonoscopy-interval recommendations were benchmarked against guideline-defined reference intervals for diagnostic agreement.	Agreement percentage, Fleiss' kappa, McNemar test.
Chang, 2024	Auxiliary Diagnosis	QUADAS-2	Post-colonoscopy surveillance recommendations were compared against USMSTF panel consensus, consistent with diagnostic accuracy design.	Agreement rate, Fleiss' kappa.
Alzaid, 2024	Information Extraction	QUADAS-2	Standardized extraction of pathology reports was evaluated with AUROC, homologous to diagnostic accuracy evaluation.	Accuracy, Kappa, AUROC.
Johnson, 2025	Information Extraction	QUADAS-2	Extraction of binary diagnostic labels (dysplasia, HGD/CRC) was evaluated via sensitivity/specificity against pathology results.	F1-score, PPV, NPV, sensitivity, specificity, MCC.
Chizhikova, 2025	Information Extraction	QUADAS-2	Automated extraction of TNM staging from radiology reports was evaluated against surgical-pathology reference standards.	Accuracy, macro F1-score, precision, recall.
Zeng, 2025	Treatment Decision	QUADAS-2	Binary recommendation (need for additional surgery) was compared against pathology-derived guideline standards, forming a diagnostic accuracy design.	Accuracy; guideline consistency proportion.
Chatziisaak, 2025	Treatment Decision	QUADAS-2	Categorical treatment recommendations were compared against MDT consensus to calculate concordance, consistent with diagnostic accuracy design.	Full/partial/inconsistent concordance, chi-square test.
Kaiser, 2024	Treatment Decision	QUADAS-2	Single-choice 'next management' answers were graded against NCCN guideline reference answers on a defined accuracy scale.	Accuracy score (1–4 scale), consistency, verbosity.
Garg, 2026	Treatment	QUADAS-2	Structured surveillance-interval recommendations were	Case-level accuracy, Cohen's κ,

	Decision		benchmarked against USMSTF/ACG/ASGE reference standards for case-level accuracy.	Fleiss' κ; ADR, SSLDR, cecal intubation rate, bowel-prep adequacy.
Gorelik, 2023	Knowledge QA	QUADAS-2	Guideline-based next-step recommendations were compared against reference guideline standards to calculate adherence and accuracy.	Guideline adherence, accuracy, Fleiss' kappa.
Zhou, 2024	Knowledge QA	QUADAS-2	Closed-ended (yes/no) answers were benchmarked against NCCN guideline reference answers to calculate binary accuracy.	Accuracy (vs NCCN guidelines).
Atarere, 2024	Knowledge QA	QUADAS-2	Response appropriateness was benchmarked against predefined reference standards drawn from established screening guidelines.	Appropriateness rating (reliable-appropriate / reliable-inappropriate / unreliable).
Lim, 2024	Knowledge QA	QUADAS-2	Colonoscopy-interval recommendations were evaluated against guideline-defined reference intervals for accuracy and hallucination.	Correct interval percentage, hallucination rate.
Peng, 2024	Knowledge QA	ROBINS-I	LLM responses to medical questions were rated by experts as an educational intervention, without a single binary reference standard.	Accuracy, comprehensiveness scores.
Emile, 2023	Knowledge QA	ROBINS-I	Expert-rated appropriateness of 38 CRC answers served as a process-intervention outcome rather than a dichotomous diagnostic index.	Expert-consensus appropriateness; consistency with ASCRS guidelines.
Kepez, 2024	Knowledge QA	ROBINS-I	Patient-information quality was scored with multidimensional Likert instruments, typical of a non-randomized intervention evaluation.	DISCERN, GQS, JAMA criteria, Flesch-Kincaid readability, SAM, HITS, VPI, HONcode.

Wang, 2024	Knowledge QA	ROBINS-I	Comprehensibility, scientific validity and satisfaction Likert scores constitute process outcomes of an informational intervention.	7-point Likert scale (comprehensibility, scientificity, satisfaction).
Zhang, 2025	Knowledge QA	ROBINS-I	Comparative scoring of AI-generated educational text was assessed as a content-generation intervention without a single binary reference.	Accuracy, clarity, rigor scores (1–10).
Maida, 2025	Knowledge QA	ROBINS-I	Expert and patient Likert ratings evaluated patient-education output as a clinical communication intervention.	Expert scores (accuracy, completeness, clarity); patient scores (completeness, clarity, trust).
Maida, 2024	Knowledge QA	ROBINS-I	Expert and patient scoring of CRC screening responses reflects an educational intervention without a binary reference standard.	Accuracy, completeness, clarity scores (Likert scale).
Hu, 2025	Knowledge QA	ROBINS-I	Likert-scored clarity and completeness of post-operative consultations represent process outcomes of an AI-based educational intervention.	Accuracy, completeness, clarity scores (Likert scale).
Horesh, 2025	Treatment Decision	ROBINS-I	Recommendation concordance with MDT decisions was rated on multi-level Likert scales, lacking a single binary reference standard.	Consistency with MDT decisions (1–5), reasonableness (1–5), inter-rater reliability (Cohen's κ).
Schmutz, 2025	Treatment Decision	ROBINS-I	Molecular tumor-board recommendation quality was compared against human experts on continuous indices, fitting a non-randomized intervention design.	Recommendation type, information density, consistency (Fleiss' kappa), LoE, time efficiency.
Zeng, 2025	Treatment	ROBINS-I	Generated geographically tailored recommendations as a	Correct / partially correct /

	Decision		simulated workflow intervention, lacking a single binary answer.	incorrect proportions; descriptive statistics.
Qu, 2026	Treatment Decision	ROBINS-I	Multi-role simulated MDT classification was assessed for intra-model agreement and expert concordance, not against a binary diagnostic standard.	Intra-model agreement; expert-model concordance, Cohen's κ.
Yu, 2025	Information Extraction	ROBINS-I	Multi-strategy prompting for structured extraction was evaluated as an applied workflow intervention across heterogeneous models and tasks.	Precision, recall, F1-score, accuracy.
Kim JS, 2025	Information Extraction	ROBINS-I	Preoperative staging extraction from CT/MRI reports with reasoning output was evaluated as a workflow-oriented extraction intervention.	Lesion location accuracy; T/N/M stage accuracy; overall TNM accuracy.
Yang, 2025	Scientific Research	ROBINS-I	Natural-language queries driving downstream TP53-pathway genomic analysis constitute a research workflow intervention, not a diagnostic index test.	p-values, hazard ratios, odds ratios.
Diaz, 2025	Scientific Research	ROBINS-I	Precision-medicine survival analyses via AI-agent queries represent a research intervention rather than a diagnostic accuracy study.	p-values, odds ratios, survival rates.
Sehgal, 2025	Aided Nursing	ROBINS-I	Effect of AI-generated messages / chatbot on screening intent was evaluated as a behavioral intervention with pre-post outcomes.	Intent score change (0–100), Cohen's d, p-values, OR, Flesch-Kincaid readability.
