# Supplementary material for: Large Language Models in Colorectal Cancer Care and Clinical Decision Support: Systematic Review
Source: J Med Internet Res. 2026 May 21;28:e89862. doi: 10.2196/89862 (PMC13193707; doi:10.2196/89862)
Supplement: Multimedia Appendix 3 [file jmir-v28-e89862-s003.pdf]

Multimedia Appendix 3 : Documentation of Framework-Preserving Adaptations to Quality Appraisal Tools

---

1. Overview

This appendix documents the specific adaptations made to three established quality appraisal tools—PROBAST, QUADAS-2, and ROBINS-I—for application to studies evaluating large language models (LLMs) in colorectal cancer (CRC). All adaptations were performed by an oncology expert at the chief physician level (QF), in strict adherence to the core assessment principles of each tool.

Adaptations were necessary because LLM evaluation studies differ from conventional clinical trials in several important respects: the "intervention" is a computational tool rather than a pharmacological or surgical procedure; outcome measures are predominantly technical performance metrics (e.g., accuracy, F1-score); and study designs are frequently retrospective with limited reporting of blinding, randomization, or pre-specified endpoints.

Three guiding principles governed all adaptations:

- **Preservation of core logic:** No core bias assessment domain or dimension was removed or substantively altered.
- **Transparency:** All adaptations are documented below with explicit rationale.
- **Reproducibility:** The adapted tools retain sufficient detail to permit independent replication.

2. PROBAST (Prediction Model Risk of Bias Assessment Tool)

Applied to: 2 predictive modeling studies (Kim et al, 2025; Yang X et al, 2025).

2.1 Removal of pre-process modules

The original PROBAST includes preliminary modules for specifying review questions, classifying prediction model types, and conducting multiple assessment rounds for a single model. These were removed because the PICO framework and prediction model classification had already been defined in the Methods section. All included prediction model studies addressed the same clinical context (CRC), rendering repeated classification redundant.

2.2 Streamlining of signalling questions

Signalling questions containing extended examples and restrictive explanations were condensed to retain only the core assessment question, without altering their evaluative intent. Two items were removed: (a) "whether predictor selection based on univariate analysis was avoided" and (b) "whether predictor weights in the final model were consistent with multivariate analysis results." The included retrospective prediction model studies did not report sufficient detail to permit meaningful assessment of these items. Their removal does not affect the overall judgment of any core bias domain.

2.3 Structural consolidation

The two independent sub-modules for "risk of bias" and "applicability" under each domain were merged

into a unified structure integrating signalling questions, domain-level bias ratings, and applicability ratings. Separate dual columns for model development and validation datasets were removed, as these were reported separately in all included studies. Columns for rating rationales were removed from the table; all rationales are presented in the Quality Appraisal subsection of the Results.

2.4 Addition of identification and summary columns

Columns for basic study information (author, year, model name) and overall risk of bias and applicability judgments were added to ensure completeness and traceability of study-level assessment records.

3. QUADAS-2 (Quality Assessment of Diagnostic Accuracy Studies–2)

Applied to: 17 diagnostic accuracy studies.

3.1 Removal of pre-process requirements

The original QUADAS-2 requires specifying the review question and drawing individual study flow diagrams as preliminary steps. These were removed because the review question is defined in the Methods section and the study selection process is presented in the PRISMA flow diagram (Figure 1). Retaining these modules in the assessment table would constitute duplicate reporting.

3.2 Structural consolidation

The separate risk of bias and applicability sub-modules under each domain were merged, and columns for item-level rating rationales were removed. Rating rationales are summarized in the Results section. The revised table focuses on core rating outcomes to improve readability and conciseness.

3.3 Addition of identification and summary columns

Columns for basic study information and an overall judgment column were added to ensure completeness, consistent with the assessment process described in the Methods.

4. ROBINS-I (Risk of Bias in Non-randomized Studies of Interventions)

Applied to: 15 non-randomized intervention studies evaluating LLM application effects, including information extraction and knowledge-based tasks.

4.1 Removal of pre-process modules

The original ROBINS-I includes extensive preliminary modules: listing confounding factors, defining target trials, distinguishing intention-to-treat from per-protocol effects, and preliminary screening for critical risks. These were removed because core confounding factors were pre-defined in the Methods section, and all included studies were non-randomized studies of a homogeneous intervention type (LLM application in CRC care). Retaining these preparatory modules would introduce unnecessary operational complexity without altering the core bias assessment logic.

4.2 Restoration of the classic 7-domain framework

The ROBINS-I version 2 removed the domain “bias due to deviations from intended interventions.” This domain was restored because deviations from intended LLM use—such as variations in prompt

formulation, model version changes, or inconsistent input formatting—are clinically relevant sources of bias in the included studies. This restoration returns the tool to the classic 7-domain assessment framework and ensures comprehensive coverage of intervention-related bias.

4.3 Streamlining of signalling questions

Signalling questions with complex branching logic were simplified to retain only the core evaluative content. Items requiring information not reported in the included studies (e.g., negative control assessment, prediction of bias direction, sensitivity analysis details) were removed because the retrospective study designs did not provide assessable information. Item numbering was unified to replace the original skipped numbering and branching structure, improving consistency across assessors.

4.4 Simplification of the rating scale

The "Critical risk" rating level was removed. All included studies had passed pre-specified inclusion and exclusion criteria; no study was judged to be at critical risk of bias. The rating scale was therefore simplified to four levels: Low, Moderate, Serious, and No Information (NI). The complex algorithmic bias judgment in the original tool was replaced by the judgment logic described in the Methods section; only final domain-level and overall ratings are presented in the table.

4.5 Addition of identification and summary columns

Columns for basic study information and an overall bias judgment column were added, consistent with the approach applied to PROBAST and QUADAS-2.

5. Summary

All adaptations described above are framework-preserving optimizations tailored to the specific research context of this systematic review. No core bias assessment domain or signalling question with substantive evaluative content was removed from any tool. The principal modifications involved:

- Removing preparatory modules already addressed in the Methods section of the manuscript.
- Consolidating sub-modules and removing redundant documentation columns to improve table readability.
- Streamlining signalling questions for which the included literature did not provide assessable information.
- Adding study identification and summary columns for completeness and traceability.

The adapted tools were applied to all 34 included studies. Assessment was conducted independently by two reviewers (JL, HT), with disagreements resolved by a third reviewer (QF). The assessment process is fully reproducible, and all results are presented transparently in the main text (Results → Quality Appraisal) and supplementary materials (Multimedia Appendix 1; Supplementary Table 1).

References

Whiting PF, Rutjes AW, Westwood ME, et al. QUADAS-2: a revised tool for the quality assessment of diagnostic accuracy studies. *Ann Intern Med*. 2011;155(8):529–536.

Moons KGM, Wolff RF, Riley RD, et al. PROBAST: a tool to assess risk of bias and applicability of prediction model studies. *Ann Intern Med*. 2019;170(1):51–58.

Sterne JAC, Hernán MA, Reeves BC, et al. ROBINS-I: a tool for assessing risk of bias in non-randomised studies of interventions. *BMJ*. 2016;355:i4919.
