# Supplementary material for: Large Language Models in Colorectal Cancer Care and Clinical Decision Support: Systematic Review
Source: J Med Internet Res. 2026 May 21;28:e89862. doi: 10.2196/89862 (PMC13193707; doi:10.2196/89862)
Supplement: Multimedia Appendix 4 [file jmir-v28-e89862-s004.pdf]

Multimedia Appendix 4: Prompt Engineering Strategies and Application Scenarios in the Included Studies

Prompt Strategy	n	Studies	Typical Application Scenarios
Instruction templates / Role prompting	22	Lim, 2024; Diaz, 2025; Chang, 2024; Zhang, 2025 ; Keez, 2024; Amini, 2025; Chatziisaak, 2025; Yang, 2025; Yang, 2025; Ding, 2025; Gorelik, 2023; Massimi, 2025; Schmutz, 2025; Wang, 2025; Johnson, 2025; Alzaid, 2024; Sehgal, 2025; Zeng, 2025; Garg, 2026; Qu, 2026; Kim, 2025	Diagnostic support; Clinical decision support; Information extraction; Patient education; MDT treatment decision simulation; TNM staging extraction from imaging reports; post-polypectomy surveillance recommendations
Zero-shot learning	8	Horesh, 2025; Peng, 2024; Wang, 2024; Hu, 2025; Liu, 2024; Zhou, 2024; Maida, 2025; Garg, 2026	Knowledge-based Q&A; Baseline performance evaluation
Few-shot learning	6	Ferber, 2024]; Yu, 2025; Zeng, 2025; Peng, 2024; Zhou, 2024; Garg, 2026	Pathology image classification; Entity extraction from clinical reports
Fine-tuning	3	Chizhikova, 2025; Kim, 2025; Yang, 2025	TNM staging; Predictive modeling
Hybrid / Advanced (CoT, RAG, multi-strategy)	4	Sehgal, 2025; Yu, 2025; Zeng, 2025; Garg, 2026	Personalized messaging; Multi-entity extraction; Guideline-based decision support; chain-of-thought reasoning with JSON schema enforcement for surveillance guideline application
Not explicitly described	5	Atarere, 2024; Kaiser, 2024; Emile, 2023; Maida, 2025; Maida, 2025	General CRC knowledge evaluation; Patient education

Note: Some studies employed multiple strategies and appear in more than one category. CoT, chain-of-thought; RAG, retrieval-augmented generation; Q&A, question and answer; MDT, multidisciplinary team; TNM, tumor-node-metastasis.
