# Supplementary material for: Large Language Models in Colorectal Cancer Care and Clinical Decision Support: Systematic Review
Source: J Med Internet Res. 2026 May 21;28:e89862. doi: 10.2196/89862 (PMC13193707; doi:10.2196/89862)
Supplement: Checklist 1 [file jmir-v28-e89862-s005.pdf]

Appendix 2: PRISMA 2020 Search Strategy Extension (PRISMA-S) Checklist

Section/Topic	Item	Checklist Item	Location in Manuscript	Notes
**INFORMATION SOURCES AND METHODS**				
Database name	1	Name each individual database searched, stating the platform for each.	Methods → Information Sources; Supplementary Appendix 1	All 6 databases listed. Recommend adding the platform after each (e.g., PubMed (MEDLINE); Embase via Embase.com; CINAHL via EBSCOhost; Web of Science via Clarivate; Scopus via Elsevier; Cochrane Library via Wiley).
Multi-database searching	2	If databases were searched simultaneously on a single platform, state the platform and list the databases.	Methods → Information Sources	Explicitly reported as not used: "Each database was searched individually."
Study registries	3	List any study registries searched.	Methods → Search Strategy (PRISMA-S statement paragraph)	Reported as not applicable, with justification.
Online resources and browsing	4	Describe any online or print source purposefully searched or browsed (e.g., websites, conference proceedings, preprints).	Methods → Search Strategy (PRISMA-S statement paragraph)	Now explicitly reported: no grey literature, websites, conference proceedings, or preprint servers searched, with justification.
Citation searching	5	Indicate whether cited/citing references were examined and describe methods used.	Methods → Search Strategy (PRISMA-S statement paragraph)	Manual reference-list screening of included studies reported; forward citation tracking explicitly not used.

Contacts	6	Indicate whether additional studies or data were sought by contacting authors, experts, manufacturers, or others.	Methods → Eligibility/Selection; Methods → Search Strategy (PRISMA-S statement paragraph)	Both uses (contact for missing data) and non-use (no contact for identifying new studies) reported.
Other methods	7	Describe any additional information sources or search methods used.	Methods → Search Strategy (PRISMA-S statement paragraph)	Now explicitly reported: PubMed Related Articles, personal files, and database-internal recommendation tools were not used.
**SEARCH STRATEGIES**				
Full search strategies	8	Include the search strategies for each database, copied and pasted exactly as run.	Supplementary Appendix 1	Complete and reproducible for all 6 databases.
Limits and restrictions	9	Specify that no limits were used, or describe any limits applied and provide justification.	Supplementary Appendix 1 (Embase line #11); not in Methods	Embase applied year (2022–2026) + human + article-type limits but Methods does not mention or justify them. Recommend adding one sentence in Methods stating these Embase-specific limits and the rationale.
Search filters	10	Indicate whether published search filters were used (as designed or modified), and if so, cite them.	Methods → Information Sources	Explicitly reported: "No published search filters were applied."
Prior work	11	Indicate when search strategies from other reviews were adapted or reused, citing them.	Methods → Search Strategy (PRISMA-S statement paragraph)	Now explicitly reported: strategy was developed de novo by the team, not adapted from prior reviews.
Updates	12	Report the methods used to update the search(es) (e.g.,	Methods → Search Strategy; Registration &	Updating to April 7, 2026 reported.

		rerunning, email alerts).	Protocol section	
Dates of searches	13	For each search strategy, provide the date the last search occurred.	Abstract; Methods → Information Sources; Supplementary Appendix 1	March 1, 2026 reported for every database.
**PEER REVIEW**				
Peer review	14	Describe any search peer review process.	Methods → Search Strategy (PRISMA-S statement paragraph)	Now explicitly reported: no formal external PRESS peer review; strategy was cross-checked internally by team members.
MANAGING RECORDS				
Total records	15	Document the total number of records identified from each database and other sources.	Results → Study Selection; Figure 1 (PRISMA flow diagram)	Per-database totals reported（PubMed 4,047 ; Embase 1,423 ; Web of Science 3,061 ; Cochrane 43 ; Scopus 43 ; CINAHL 263）
Deduplication	16	Describe the processes and any software used to deduplicate records.	Methods → Selection Process	EndNote X9.3.3 automated deduplication + manual verification reported.
